# spaLLM: enhancing spatial domain analysis in multi-omics data through large language model integration

**DOI:** 10.1093/bib/bbaf304

**Published:** 2025-07-03

**Authors:** Longyi Li, Liyan Dong, Hao Zhang, Dong Xu, Yongli Li

**Affiliations:** Key Laboratory of Symbolic Computation and Knowledge Engineering of Ministry of Education, College of Computer Science and Technology, Jilin University, 2699 Qianjin Street, Changchun 130012, Jilin, China; Key Laboratory of Symbolic Computation and Knowledge Engineering of Ministry of Education, College of Computer Science and Technology, Jilin University, 2699 Qianjin Street, Changchun 130012, Jilin, China; Key Laboratory of Symbolic Computation and Knowledge Engineering of Ministry of Education, College of Computer Science and Technology, Jilin University, 2699 Qianjin Street, Changchun 130012, Jilin, China; Department of Electrical Engineering and Computer Science, Christopher S. Bond Life Sciences Center, University of Missouri, 1201 Rollins Street, Columbia, MO 65211, United States; School of Information Science and Technology, Northeast Normal University, 5268 Renmin Street, Changchun 130117, Jilin, China

**Keywords:** spatial domain, spatial multi-omics, large language model, spatial resolved transcriptomics, graph neural network

## Abstract

Spatial multi-omics technologies provide valuable data on gene expression from various omics in the same tissue section while preserving spatial information. However, deciphering spatial domains within spatial omics data remains challenging due to the sparse gene expression. We propose spaLLM, the first multi-omics spatial domain analysis method that integrates large language models to enhance data representation. Our method combines a pre-trained single-cell language model (scGPT) with graph neural networks and multi-view attention mechanisms to compensate for limited gene expression information in spatial omics while improving sensitivity and resolution within modalities. SpaLLM processes multiple spatial modalities, including RNA, chromatin, and protein data, potentially adapting to emerging technologies and accommodating additional modalities. Benchmarking against eight state-of-the-art methods across four different datasets and platforms demonstrates that our model consistently outperforms other advanced methods across multiple supervised evaluation metrics. The source code for spaLLM is freely available at https://github.com/liiilongyi/spaLLM.

## Introduction

Spatially resolved omics technologies represent an emerging field that enables precise localization of gene expression while maintaining tissues’ structural integrity. By combining sequencing data with spatial coordinates, these technologies map molecular profiles to precise tissue locations, providing essential context for studying cellular functions in their native microenvironment. The field now encompasses both single-omics approaches on tissue sections (such as 10× Visium [1], Slide-seq [2], Slide-seq V2 [3], LCM-seq [4], and Stereo-seq [5]) and advanced multi-omics platforms that profile the same tissue section (such as DBiT-seq [6], 10× Genomics Xenium [7], SPOTS [8], Stereo-CITE-seq [9], spatial ATAC–RNA-seq and CUT&Tag-RNA-seq [10], Spatial-CITE-seq [11], MISAR-seq [12], SM-Omics [13], MiP-seq [14], and tri-omics [15]). Each technique offers unique advantages for analyzing tissue heterogeneity and cellular functions, advancing our understanding of cellular interactions and tissue organization complexity.

In spatially resolved omics analysis, spatial domains represent distinct clusters within a tissue or organism that exhibit specific molecular characteristics and functions. Identifying and characterizing these domains constitutes a fundamental and critical downstream bioinformatics task. Various existing methods typically employ deep learning approaches, such as variational autoencoder [16], Transformer [17], and graph neural network (GNN) [18, 19], to integrate multi-modal biological information. Recent methodological advances have enhanced spatial domain analysis through diverse approaches: ConGI [20] combines gene expression matrices with histopathological images through contrastive learning; spatial clustering via geometric deep learning (SCGDL) [21] leverages GNN to process feature and adjacency matrices; spatially resolved transcriptomics multi-view graph convolution network (STMGCN) [22] employs dual graph building approaches using cosine similarity and Euclidean distance; SpaNCMG [23] utilizes both *k*-nearest-neighbor (KNN) algorithm and *r*-radius graph building methods; and stCluster [24] integrates comparative learning with multi-task learning. With the emergence of spatial multi-omics technologies, models like SpatialGlue [25] have been developed to evaluate the relative importance of spatial features versus gene expression and different types of omic data using an attentional approach for multi-modal information fusion.

While scRNA-seq data provide sufficient information for cell type inference and clustering [26], spatial multi-omics data typically offer limited gene expression [27]. Current spatial omics technologies face two major challenges: limited resolution below the single-cell level and low capture efficiency across modalities. Achieving higher resolution with improved sensitivity and specificity would significantly advance the field [28]. Additionally, current methods rely solely on numerical information from spot-gene expression matrices for similarity calculations without incorporating gene-specific biological information. Large language models (LLMs) have emerged as a promising solution to these challenges, offering powerful computational capabilities for biological data analysis.

LLMs have recently gained recognition in bioinformatics as a powerful and versatile approach. These models can obtain rich knowledge representations through pre-training on large-scale datasets, providing insightful characterizations of cellular states. Notable examples include Geneformer [29], pre-trained on approximately 30 million cells using a self-supervised approach, and other ultra-large-scale scRNA-seq-based models such as scBERT [30], scGPT [31], and scFoundation [32]. These models enable efficient data integration, feature learning, and phenotype prediction while facilitating multiple downstream tasks. However, despite these advances, applying LLMs to enhance spatial omics data characterization remains unexplored. Furthermore, while methods for simultaneous measurement of multiple omics exist [15], computational models designed explicitly for multi-omics data integration and analysis remain scarce.

To address these challenges, we propose a **spa**tial domain analysis method using **l**arge **l**anguage **m**odels (spaLLM). Our approach integrates LLM-generated representations from single-cell transcriptome data with spatially resolved multi-omics data. By fusing multiple representations with LLM embeddings, spaLLM reduces feature engineering complexity [33] and enhances model informativeness. Specifically, we leverage scGPT [31] and GNN to encode spatial omics data into latent representations, which are then aggregated through an attention mechanism for downstream clustering and visualization. Comprehensive evaluation against eight state-of-the-art methods demonstrates that spaLLM consistently outperforms existing approaches across multiple datasets and platforms.

## Materials and methods

### Overview of the method

**Figure 1:**
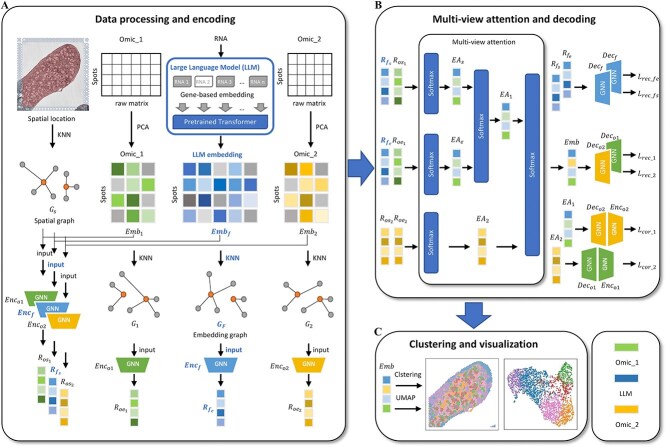
Overall architecture of spaLLM, showing (A) data processing, LLM embedding of the gene expression matrix, and construction of spatial and embedding graphs for GNN encoding, (B) aggregation of the six resulting tensors via a multi-view attention layer to produce the final latent representation, and (C) downstream spatial domain deciphering and uniform manifold approximation and projection visualization using the final latent representation.

SpaLLM integrates spatially resolved multi-omics data using LLM and GNN to decipher spatial domains. SpaLLM's overall data flow is shown in Fig. 1. The original omics data matrices of two omics are ${X}_1=\left\{{x}_{11},{x}_{12},\dots, {x}_{1N}\right\}\in{\mathbb{R}}^{N\times{D}_1}$ and ${X}_2=\left\{{x}_{21},{x}_{22},\dots, {x}_{2N}\right\}\in{\mathbb{R}}^{N\times{D}_2}$, where *N* denotes number of the spots, ${D}_1$ and ${D}_2$ represent the numbers of genes, peaks, or proteins in the two omics data. And let $P=\left\{{p}_1,{p}_2,\dots, {p}_N\right\}\in{\mathbb{R}}^{N\times 2}$ denote the spot coordinate matrix. SpaLLM combines the scRNA-seq-based representations generated by LLM with spatially resolved multi-omics data, reducing the complexity of feature engineering and making the model more informative for learning. Specifically, spaLLM first encodes spatial transcriptomic data and spatial omics data with the help of LLM and GNN to obtain latent representations. Next, the latent representation is obtained by aggregating multiple representations by means of attention. We use the final embeddings for downstream clustering, achieving competitive results across multiple datasets. Our method more accurately delineates spatial domains than existing spatial multi-omics approaches.

### L‌LM embedding

Each cell contains a large number of genes. LLMs, by learning the gene expression information from a vast number of cells, can provide a more precise latent representation for each cell. scGPT [31] is a large-scale pre-trained LLM based on scRNA-seq data with a gene vocabulary of size 60 697, capable of performing a variety of downstream tasks. Current spatial omics methods use only numerical spot–gene counts to calculate the similarity between spots, without incorporating the specific information provided by the genes themselves. With the aim of using scRNA-seq data to bridge the gap in spatial transcriptomics, we input the complete gene expression vector from each spot ${X}_1$ into scGPT, yielding embedding ${Emb}_f=\left\{{E}_{f_1},{E}_{f_2},\dots, {E}_{f_N}\right\}\in{\mathbb{R}}^{N\times{D}_f}$. ${D}_f$ denotes the embedding dimension of each spot. Our experiments demonstrate that scGPT produces embeddings with superior representation and stability (Supplementary Fig. S1). In this way, each spot gains an additional representation, enhancing the sensitivity and specificity of spatial omics data, which do not achieve single-cell resolution. Furthermore, the integration of multiple representations provides more information and features for the identification of spatial domains.

### Graph construction

To fuse information across multiple modalities, we built the multi-view graph structure, which includes a spatial graph and three embedding graphs. They are a spatial graph ${G}_s=\left(V,{E}_s\right)$, an omic-specific embedding graph ${G}_1=\left(V,{E}_1\right)$, another omic-specific embedding graph ${G}_2=\left(V,{E}_2\right)$, and an embedding graph for LLM embedding ${G}_F=\left(V,{E}_F\right)$. A spatial graph purely considers the spatial correlations between spots. For a tissue slice with multi-omics data, the distribution of spot locations is fixed and consistent across the different omics. When constructing a spatial graph, each spot is connected to the $k$ nearest spots in Euclidean space. The calculation method is as follows:


(1) \begin{equation*} {A}_s\left(i,j\right)=\sigma \left({\left\Vert{p}_i-{p}_j\right\Vert}_2^2\right) \end{equation*}

where ${A}_s$ is the adjacency matrix of spatial graph, $\sigma \left(\bullet \right)$ is a function, and $i$ and $j$ are any two distinct spots in the set. If the Euclidean distance between ${p}_i$ and ${p}_j$ is among the smallest ${k}_1$, $\sigma \left(\bullet \right)$ returns a value of 1; otherwise, it returns 0.

Given that the difference in spatial proximity does not necessarily directly represent the difference in spot type, we apply the KNN [34] algorithm to the downsized feature expression matrix, generating an embedding graph based on omics information. The edges of the embedding graph represent the proximity of omics features between two spots. Specifically, for the original spatial omics data ${X}_1$ and ${X}_2$, we use principal component analysis (PCA) [35] to downsize the matrix to ${Emb}_1=\left\{{E}_{11},{E}_{12},\dots, {E}_{1D}\right\}\in{\mathbb{R}}^{N\times D}$ and ${Emb}_2=\left\{{E}_{21},{E}_{22},\dots, {E}_{2D}\right\}\in{\mathbb{R}}^{N\times D}$, where $D$ denotes the dimension of the embeddings. For the LLM embedding ${Emb}_f$, the omics embedding ${Emb}_1$ and ${Emb}_2$, we compute Pearson correlations between spot embeddings. For the embedding of $i$ and $j$, ${E}_i=\left({x}_1,{x}_2,\dots, {x}_D\right)$, and ${E}_j=\left({y}_1,{y}_2,\dots, {y}_D\right)$, the calculation method is as follows:


(2) \begin{equation*} {\displaystyle \begin{array}{c}{A}_e\left(i,j\right)=\sigma \left(r\left({E}_i,{E}_j\right)\right)\end{array}} \end{equation*}

(3) \begin{equation*} {\displaystyle \begin{array}{c}r=\frac{\sum_{i=1}^n\left({x}_i-\overline{x}\right)\left({y}_i-\overline{y}\right)}{\sqrt{\sum_{i=1}^n{\left({x}_i-\overline{x}\right)}^2}\sqrt{\sum_{i=1}^n{\left({y}_i-\overline{y}\right)}^2}}\end{array}} \end{equation*}

where ${A}_e$ is the adjacency matrix of embedding graph, $\sigma \left(\bullet \right)$ is a function. If the Pearson correlation coefficient between ${E}_i$ and ${E}_j$ is among the top ${k}_2$ highest values, $\sigma \left(\bullet \right)$ returns a value of 1; otherwise, it returns 0. We set ${k}_1=3$ and ${k}_2=20$ empirically.

### Encoder GNNs

To encode different input data into latent space, we utilize an unsupervised GNN to perform encoding for multi-modal data. This approach captures features in gene expression while simultaneously learning the spatial topological structure [36]. The encoder learns latent representations of input features through three layers of linear transformation, each layer employs linear transformation, adjacency-based message passing, and a non-linear activation function to project features into latent spaces. The encoder is propagated as follows:


(4) \begin{equation*} {\displaystyle \begin{array}{c}{Z}_m^{(1)}=\mathrm{\sigma} \left({A}_m{Z}_m^{(0)}{W}^{(0)}\right)=\mathrm{\sigma} \left({A}_m\bullet \left(X{W}^{(0)}\right)\right)\end{array}} \end{equation*}

(5) \begin{equation*} {\displaystyle \begin{array}{c}{Z}_m^{(2)}=\mathrm{\sigma} \left({A}_m{Z}_m^{(1)}{W}^{(1)}\right)\end{array}} \end{equation*}

(6) \begin{equation*} {\displaystyle \begin{array}{c}{Z}_m^{(3)}={A}_m{Z}_m^{(2)}{W}^{(2)}\end{array}} \end{equation*}

where $m$ represents different modality including LLM embedding and multi-omics embedding. ${A}_m$ is the input adjacency matrix, ${Z}_m^{(0)}=X$ represents the feature embeddings derived from the original omics data matrix for each spot, obtained either through LLM or by applying PCA, $W$ is the weight matrix, and $\sigma \left(\bullet \right)$ is the activation function, typically a ReLU, introducing non-linearity to the transformation. For the embedding ${Emb}_f$ obtained from LLM, we input ${Emb}_f$ and the spatial graph ${G}_s$ into the encoder ${Enc}_f$, and similarly input ${Emb}_f$ and the embedding graph ${G}_e$ into the encoder ${Enc}_f$ as well. For the first omic embedding ${Emb}_1$ and the second omic embedding ${Emb}_2$, we input ${Emb}_1$ into the encoder ${Enc}_{o1}$ with the spatial graph ${G}_s$ and the embedding graph ${G}_1$ respectively. Similarly, we input ${Emb}_2$ into the encoder ${Enc}_{o2}$ with the spatial graph ${G}_s$ and the embedding graph ${G}_2$, respectively.

### Multi-view attention

In order to integrate spatial and gene expression information, spatial multi-omics data, and LLM embeddings, we drew inspiration from the dual-attention in SpatialGlue [25] and designed a multi-view attention mechanism. After encoding, for the LLM embedding, we got latent representation ${R}_{f_s}$ and ${R}_{f_e}$ from ${Enc}_f$, which are derived from transcriptomic data. For multi-omics data, we got ${R}_{os_1}$ and ${R}_{oe_1}$ from ${Enc}_{o1}$, which are derived from transcriptomic data, ${R}_{os_2}$ and ${R}_{oe_2}$ from ${Enc}_{o2}$, which are derived from chromatinome or proteome data, respectively (Fig. 1b). Our attention module first compresses the two or four input embeddings and concatenates them along a new dimension to form a combined representation matrix. Subsequently, the attention weights are computed through the following steps:


(7) \begin{equation*} {\displaystyle \begin{array}{c}v=\tanh \left(R\bullet{W}_{\omega}\right)\end{array}} \end{equation*}

(8) \begin{equation*} {\displaystyle \begin{array}{c}{v}_{\mu }=v\bullet{\mu}_{\omega}\end{array}} \end{equation*}

(9) \begin{equation*} {\displaystyle \begin{array}{c}{\alpha}_i^k=\mathrm{softmax}\left( v\mu +\varepsilon \right)=\frac{\exp \left(v{\mu}_i^k+\varepsilon \right)}{\sum_{k=1}^M\exp \left(v{\mu}_i^k+\varepsilon \right)}\end{array}} \end{equation*}

(10) \begin{equation*} {\displaystyle \begin{array}{c}{R}^{{\prime}}={R}^T\bullet \alpha \end{array}} \end{equation*}

where $R$ is the combined representation matrix constructed by concatenating the latent representation (with each of shape ${\mathbb{R}}^{N\times 64}$, such that $R$ is a tensor of shape ${\mathbb{R}}^{N\times M\times 64}$), ${W}_{\omega }$ and ${\mu}_{\omega }$ are weight matrices, $\varepsilon$ is a small positive constant added for numerical stability, $M$ is the number of input embeddings. The superscript $k$ is intended to denote the $k$th modality among the set of input representations. $\alpha$ denotes the collection of attention weights for all spots and modalities. Importantly, our experimental results demonstrate that the attention calculation remains robust across different initialization parameters (see Supplementary Fig. S2). The first view of the attention is designed for the LLM. We input ${R}_{f_s}$ and ${R}_{os_1}$, ${R}_{f_e}$ and ${R}_{oe_1}$ to the attention module, producing the first set of embeddings ${EA}_s$ and ${EA}_e$, respectively, along with the corresponding attention weights. The second view of the attention is designed for the spatial and feature integration. Attention is applied by inputting ${EA}_s$ and ${EA}_e$, resulting in embedding ${EA}_1$ and its associated attention weights. Similarly, we integrate ${R}_{os_2}$ and ${R}_{oe_2}$ to ${EA}_2$. Finally, in the third view of the attention, we focus on all modalities. ${EA}_1$ and ${EA}_2$ are passed into the attention module for cross-modality fusion, generating the final combined latent representation $Emb$, along with the attention weights.

### Decoder and loss function

To train the model, update the weights, and obtain better representations for the embeddings, the decoder part of spaLLM employs GNN that is symmetrical to the encoder and designs loss function specifically for the encoder. Specifically, there are loss functions designed for the LLM encoder and the multi-omics encoder. The decoder is propagated as follows:


(11) \begin{equation*} {\displaystyle \begin{array}{c}{Z}_m^{(l)}=\mathrm{\sigma} \left({A}_m{Z}_m^{\left(l-1\right)}{W}^{\left(l-1\right)}\right)\end{array}} \end{equation*}

where $l$ is the number of layers. The LLM tensor ${R}_{f_s}$ and ${R}_{f_e}$ are decoded by ${Dec}_f$. The final embedding $Emb$ and the multi-omics tensor ${EA}_1$ and ${EA}_2$ are decoded by ${Dec}_{o1}$ and ${Dec}_{o2}$ (Fig. 1b). All the loss functions utilize mean-squared error, defined as follows:


(12) \begin{equation*} {\displaystyle \begin{array}{c}{L}_{\mathrm{MSE}}=\frac{1}{N}\sum_{i=1}^N{\left\Vert{x}_i-{\hat{x}}_i\right\Vert}_F^2\end{array}} \end{equation*}

where ${x}_i$ and ${\hat{x}}_i$ are the two comparing components. For the tensor ${R}_{f_s}$, ${R}_{f_e}$, we build reconstruction loss ${L}_{rec\_ fs}$ and ${L}_{rec\_ fe}$ between the original LLM embedding and their decoded tensor, respectively. For the final latent representation $Emb$, we build reconstruction loss ${L}_{rec\_1}$ and ${L}_{rec\_2}$ between the PCA results ${Emb}_1$ and ${Emb}_2$ and its decoded tensor. For the tensor ${EA}_1$ and ${EA}_2$, we build corresponding loss according to SpatialGlue [25]. Specifically, ${EA}_1$ is firstly decoded by ${Dec}_{o2}$ and then encoded by ${Enc}_{o2}$, the corresponding loss ${L}_{cor\_1}$ is computed between the encoded tensor and ${EA}_1$. Similarly, ${EA}_2$ is decoded and encoded by ${Dec}_{o1}$ and ${Enc}_{o1}$, the corresponding loss ${L}_{cor\_2}$ is computed between the encoded tensor and ${EA}_2$. The final loss is calculated as follows:


(13) \begin{equation*} {\displaystyle \begin{array}{c}L={\alpha}_1{L}_{re{c}_1}+{\alpha}_2{L}_{re{c}_2}+{\alpha}_3{L}_{re{c}_{fs}}+{\alpha}_4{L}_{re{c}_{fe}}+{\beta}_1{L}_{co{r}_1}+{\beta}_2{L}_{co{r}_2}\end{array}} \end{equation*}

where ${\alpha}_1$, ${\alpha}_2$, ${\alpha}_3$, ${\alpha}_4$, ${\beta}_1$, and ${\beta}_2$ are the loss weights. These parameters are optimized based on grid-based hyper-parameter fine-tuning.

After training the model, we use the final combined latent representation $Emb$ to perform spatial domain deciphering by applying the mclust package in the R programming environment, which is designed for model-based clustering and classification.

## Results

### Application to MISAR-seq mouse brain dataset

**Figure 2:**
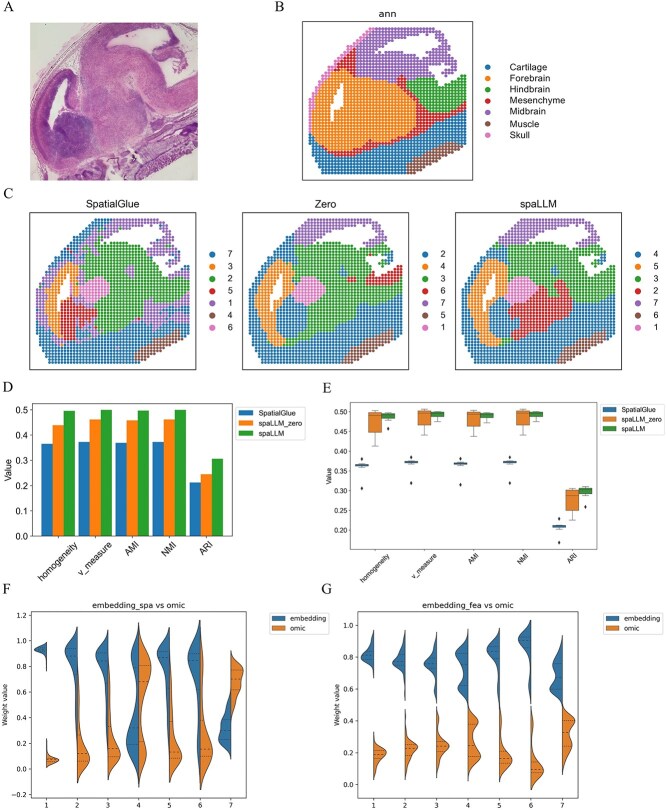
Spatial domains deciphered in the MISAR-seq mouse embryonic (E15.5) brain dataset, showing (A) H&E-stained image of the brain slice, (B) manually annotated labels, (C) spatial-domain visualizations, (D) bar chart of five supervised metrics, (E) box plots of these metrics across training epochs (median line, interquartile box, whiskers at 1.5× IQR), and (F–G) violin plots of attention weights.

MISAR-seq data and spot coordinate for the mouse embryonic E15.5 brain were obtained from https://figshare.com/articles/dataset/Spatial_genomics_datasets/21623148/5?file=42520831, following the work of Tian *et al.* [16]. The raw MISAR-seq [12] was downloaded from https://zenodo.org/records/7480069. According to the original study, labels were manually annotated using the Allen Brain Atlas and hematoxylin and eosin (H&E) images. mRNA counts were generated using Cellranger, while scATAC-seq counts were produced with ArchR (v1.0.2). The dataset comprises 1949 spots with counts for 2144 mRNAs and 47 287 peaks. During LLM embedding, 1963 mRNA sequences matched the scGPT vocabulary. Figure 2a presents the H&E staining image of the mouse E15.5 brain slice, serving as a visual reference for the spatial organization of the tissue. Figure 2b displays the manually annotated labels, which serve as ground truth for brain regions. In the current approach, SpatialGlue [25] outperforms other spatial domain analysis methods aimed at spatial multi-omics. Therefore, we first compare our method against SpatialGlue.

To evaluate the performance of our approach in spatial domain detection, Fig. 2c compares the spatial domains visualizations detected by SpatialGlue, spaLLM utilizing LLM embeddings filled with zeros, and spaLLM. The results demonstrate that spaLLM identifies more refined spatial domains closely aligned with the morphological image. In terms of region identification, spaLLM is more accurate, particularly in detecting the cartilage of the mouse brain. Figure 2d quantitatively compares the three methods across five supervised metrics: homogeneity, v-measure, adjusted mutual information (AMI), normalized mutual information (NMI), and adjusted rand index (ARI). The results indicate how well each method performs in terms of clustering and spatial domain detection, showing that spaLLM and spaLLM_zero (spaLLM utilizing embeddings filled with zeros) achieve consistently higher scores. This highlights the advantages of our GNN model architecture. Furthermore, the spaLLM model with LLM embedding as additional input consistently outperforms the spaLLM_zero model, where the embeddings are set to zero, across all metrics. This ablation study demonstrates the effectiveness of incorporating LLM embeddings. Figure 2e provides box plots illustrating the robustness of the methods over multiple training epochs, based on the five supervised metrics. In these plots, the central line denotes the median, the box represents the interquartile range, and the whiskers extend to 1.5 times the interquartile range, capturing the variability in performance. SpaLLM exhibits more stable and superior clustering across conditions.

In order to demonstrate the role of the LLM embedding and the RNA omic embedding in the final latent representation, we draw violin plots. These plots illustrate the attention weights between the LLM embedding encoded with spatial information and the omic embedding (Fig. 2f), and between the LLM embedding encoded with gene expression information and the omic embedding (Fig. 2g). Cluster 5 (pericapsular adipose tissue) shows a notably higher spatial embedding attention weight, suggesting that distinctive spatial organization and morphology drive feature extraction in this region. In contrast, Cluster 6 (capsule) and other clusters rely more on transcriptional differences, as evidenced by higher omics weights, while LLM embedding weights remain generally lower than omic embedding, indicating a modest yet positive supplementary contribution.

### Application to 10× human lymph node dataset

**Figure 3:**
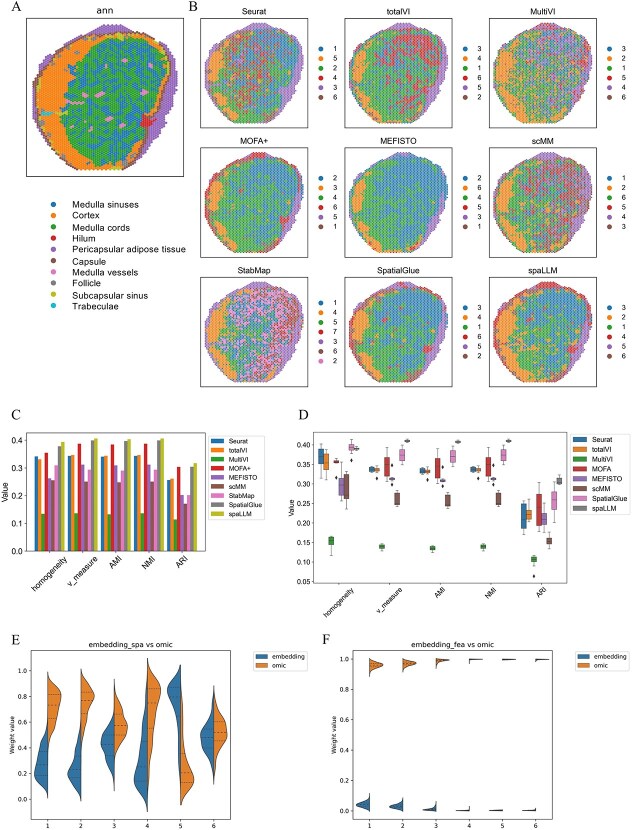
Spatial domains deciphered in the 10× human lymph node dataset, showing (A) manually annotated labels, (B) spatial domain detected by nine computational methods, (C) a bar chart of five supervised metrics, (D) box plots of these metrics, and (E–F) violin plots of attention weights.

We utilized the human lymph node dataset [25] obtained from 10× Genomics Visium CytAssist to evaluate the performance of spaLLM in spatial domain identification, comparing it with eight recently developed representative methods (i.e. Seurat [37], TotalVI [38], MultiVI [39], MOFA+ [40], MEFISTO, scMM [41], StabMap [42], and SpatialGlue [25]). The dataset was downloaded from https://zenodo.org/records/10362607 and https://drive.google.com/drive/folders/1RlU3JmHg_LZM1d-o6QORvykYPoulWWMI. This dataset contains counts for 18 085 RNAs and 31 ADTs (antibody-derived tags), which represent cell surface protein markers, across 3484 spots. During LLM embedding, 17 841 RNA sequences matched the scGPT vocabulary. Following the original study, we obtained the clustering results for each model. After training the models and obtaining latent representations, the “mclust” algorithm was applied for clustering. Figure 3a displays the manually annotated labels for the A1 slice of the human lymph node sample, which serves as a reference for identifying different regions in the tissue. These annotations serve as ground truth for method evaluation. Figure 3b showcases the spatial domains detected by nine computational methods. The spatial domain visualizations highlight how each method identifies distinct regions within the tissue. Notably, spaLLM demonstrates finer domain delineation and more accurate morphological capture than other methods. In particular, it identifies the trabeculae and subcapsular sinus with greater detail and accurately detects the hilum and capsule regions, which most other methods overlook. Moreover, spaLLM exhibits exceptional precision in delineating the cortex, with detected areas closely aligning with the labels.

Given that the dataset includes H&E-based annotations as the ground truth, we evaluate performance using supervised metrics. To quantitatively assess the clustering performance of these methods, Fig. 3c shows a bar chart of five metrics, clearly indicating that spaLLM consistently outperforms other methods. Figure 3d provides box plots of clustering performance across different cluster numbers, further demonstrating spaLLM's robustness, with tighter interquartile ranges and higher median scores in v-measure, AMI, NMI, and ARI, indicating more stable performance across different training conditions compared to the other methods. Finally, violin plots Fig. 3e represent the attention weights between the LLM embedding encoded with spatial information and the omic embedding, while Fig. 3f shows the attention weights between the LLM embedding encoded with feature information and the omic embedding. These violin plots highlight the crucial role of integrating spatial and gene expression information in enhancing the latent representations in spaLLM. The plots reveal that both the embedding_spa and omic embedding make significant contributions to the final latent representation. In Clusters 1–4, omic embedding plays a more dominant role, while in Cluster 5, embedding_spa exerts greater influence, and in Cluster 6, their contributions are nearly equal. Compared to embedding_fea, omic embedding is assigned significantly higher weights.

### Application to SPOTS mouse spleen dataset

**Figure 4:**
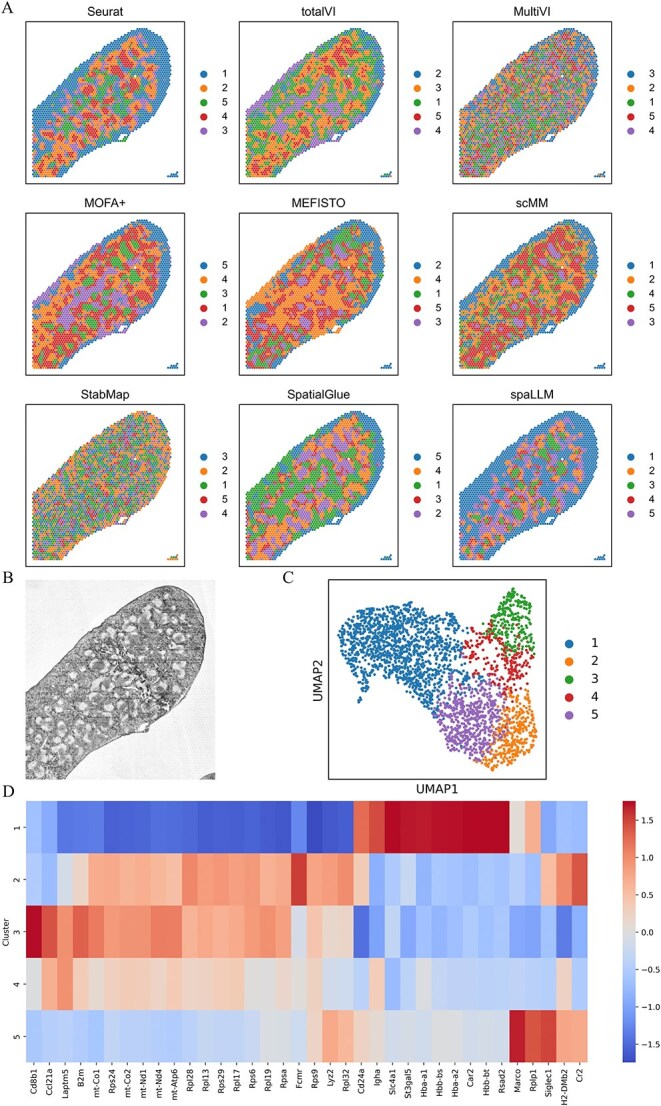
Spatial domain deciphered in the SPOTS mouse spleen dataset, showing (A) spatial domains detected by nine computational methods, (B) histology image of the sample, (C) UMAP visualization of spaLLM clustering results, and (D) top RNAs identified in each cluster.

The SPOTS mouse spleen dataset [8, 25] was downloaded from https://www.ncbi.nlm.nih.gov/geo/query/acc.cgi?acc=GSE198353 and https://zenodo.org/records/10362607. We utilized the replicate two samples, containing counts for 32 285 RNAs and 21 ADTs across 2768 spots. During the generation of the LLM embedding, 16 165 RNA sequences were matched with the scGPT’s vocabulary. Figure 4a shows the spatial domains detected by Seurat, totalVI, MultiVI, MOFA+, MEFISTO, scMM, StabMap, SpatialGlue, and spaLLM. Notably, the predictions made by spaLLM demonstrate a remarkable alignment with the morphological features observed in the histology image of the mouse spleen (Fig. 4b), indicating the model’s ability to accurately capture spatial structures. Supplementary Fig. S4 further highlights spaLLM’s superior capability in detecting more detailed spatial domains. This refinement in spatial domain identification is key to understanding intricate tissue organization, demonstrating spaLLM’s strength in spatial domain analysis. Supplementary Fig. S5 displays violin plots of the attention weight distribution, showing that the LLM embedding consistently receives substantially higher weights than the omic embedding. In the SPOTS mouse spleen dataset, this suggests that the LLM embedding is particularly critical for shaping the final latent representation. Cluster 1 shows high expression of RBC-associated genes (e.g. Slc4a1, Hba-a1, Hba-a2, and Hbb-bt), suggesting active phagocytosis of erythrocytes, a hallmark of red pulp macrophages (RpMØ). Cluster 2 exhibits marked upregulation of Fcmr and related molecules indicative of B-cell activation, while the pronounced expression of Cd8b1 in Cluster 3 implies a predominance of CD8^+^ T cells. In Cluster 5, the significant expression of MARCO together with elevated Siglec1 levels confirms its identity as marginal zone macrophages, key players in pathogen capture and innate immunity.

### Application to Spatial-CITE-seq human tonsil dataset

**Figure 5:**
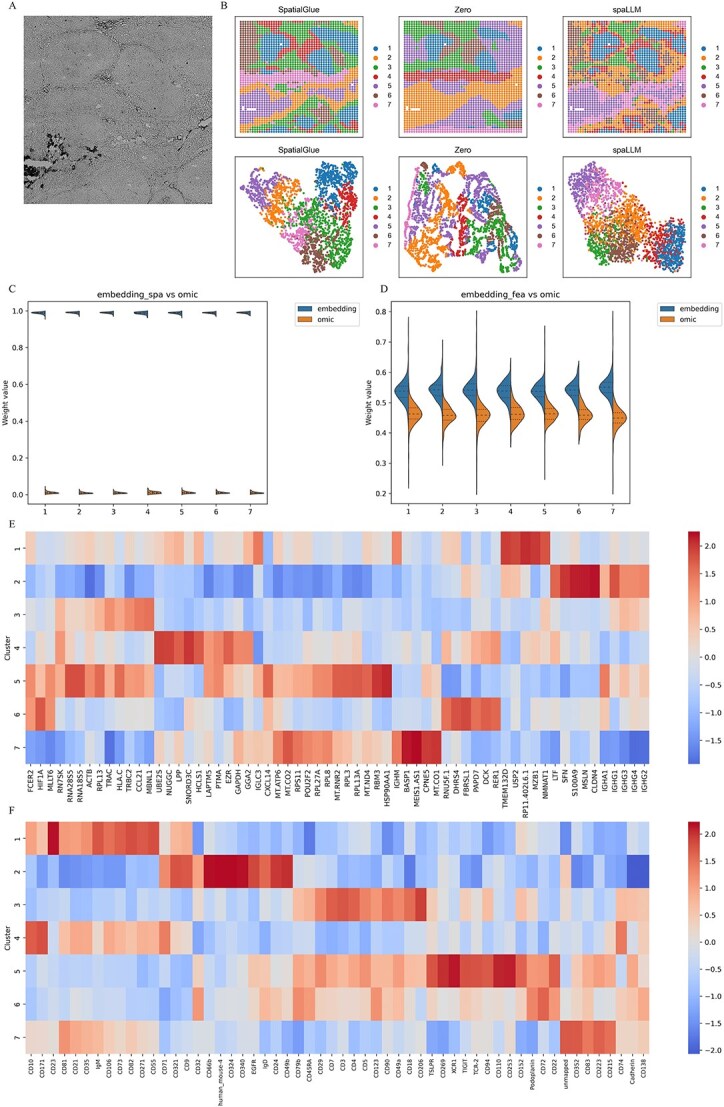
Spatial domains deciphered in the Spatial-CITE-seq human tonsil dataset, showing (A) microscope image of the tonsil slice, (B) spatial domains and UMAP visualization from SpatialGlue, spaLLM_zero and spaLLM, (C–D) violin plots of attention weights, and (E-F) top RNAs and proteins in each cluster.

We applied spaLLM to the Spatial-CITE-seq human tonsil dataset [11] to assess its capability in resolving spatial domains. This dataset contains counts for 28 417 RNAs and 283 proteins across 2492 spots. The dataset and the microscope image were downloaded from https://www.ncbi.nlm.nih.gov/geo/query/acc.cgi?acc=GSE213264 and https://figshare.com/articles/figure/figures/20723680. During the generation of the LLM embedding, 18 613 RNA sequences were matched with the scGPT’s vocabulary. Figure 5a shows the microscope image of the human tonsil slice from the Spatial-CITE-seq dataset, highlighting the morphological and biological features of the slice, such as the dark zone and light zone of the germinal center. This image provides a meaningful reference for assessing the spatial domain predictions made by different methods. Figure 5b compares spatial domains and uniform manifold approximation and projection plots from SpatialGlue, spaLLM_zero, and spaLLM. The spatial domains predicted by spaLLM exhibit a closer alignment with the morphological features seen in the microscope image, especially in dark zone and light zone, and spaLLM captures more detailed and refined spatial structures compared to the other methods. This indicates that spaLLM provides superior spatial domain resolution in this dataset. Figure 5c and d shows that the spatially encoded LLM embedding receives significantly higher, and the feature-encoded LLM embedding slightly higher, attention weights than the omic embedding, underscoring the critical role of spatial information, suggesting that feature-based embeddings play a larger role in this context. Cluster 4 (light zone) shows high levels of HCLS1, LPP, CD10, and CD71—reflecting roles in selection and adhesion—whereas Cluster 1 (dark zone) is marked by elevated RP11.402 L6.1, MZB1, NMNAT1, USP2, IgM, CD23, and CD55, indicative of active proliferation and secretion. These findings demonstrate a clear molecular distinction between the light and dark zones of the germinal center (Fig. 5e and f and Supplementary Fig. S3).

## Conclusion and discussion

In this study, we introduced spaLLM, a novel multi-omics spatial domain analysis method that integrates LLMs to overcome sparse gene coverage in spatial multi-omics datasets. By leveraging comprehensive gene-related information rather than relying solely on numerical counts, spaLLM enhances the sensitivity and specificity of high-resolution spatial analyses. Our method demonstrates robust integration capabilities, accommodating multiple spatial modalities, and shows adaptability to emerging omics technologies.

The superior performance of spaLLM across four supervised metrics and diverse datasets underscores its potential as a leading tool for spatial domain analysis, with implications for advancing spatial genomics and tissue microenvironment studies. Notably, spaLLM remains highly efficient, requiring only a few tens of seconds for embedding generation and training on a server with an Intel Xeon Silver 4210R CPU and NVIDIA RTX 3090 GPU. Our novel integration of multi-view attention and GNN effectively preserves complex spatial omics information, crucial in applications ranging from disease research to precision medicine.

Despite its robustness with sequence-based modalities, spaLLM currently lacks native support for image-based data. Future work should focus on extending spaLLM to incorporate additional modalities and applying the model across diverse tissue contexts. Furthermore, investigating optimization strategies for LLM embeddings could enhance performance, opening new avenues for spatially resolved insights in complex biological systems.

**Key Points**

- We propose spaLLM, the first multi-omics spatial domain analysis method incorporating large language model. It leverages scRNA-seq data to compensate for the relatively limited gene expression information in spatial omics, enhancing sensitivity, and specific high resolution within the modality.
- By incorporating gene-specific contextual information, our approach addresses the current limitation of methods that rely solely on numerical information to calculate spot similarity.
- Our method is capable of integrating multiple spatial omics modalities, spanning RNA, chromatin accessibility, and protein. It has the potential to adapt to emerging technologies and incorporate additional data modalities.
- Experimental results across four datasets from different sequencing platforms demonstrate that spaLLM outperforms eight state-of-the-art methods across four supervised evaluation metrics.

## Supplementary Material

Supplementary_materials_bbaf304

## Data Availability

All the data underlying this article are available in the article and in its online supplementary material.

## References

[1] Ståhl PL, Salmén F, Vickovic S. et al. Visualization and analysis of gene expression in tissue sections by spatial transcriptomics. *Science* 2016;353:78–82. 10.1126/science.aaf2403 27365449

[2] Rodriques SG, Stickels RR, Goeva A. et al. Slide-seq: a scalable technology for measuring genome-wide expression at high spatial resolution. *Science* 2019;363:1463–7. 10.1126/science.aaw1219 30923225 PMC6927209

[3] Stickels RR, Murray E, Kumar P. et al. Highly sensitive spatial transcriptomics at near-cellular resolution with slide-seqV2. *Nat Biotechnol* 2021;39:313–9. 10.1038/s41587-020-0739-1 33288904 PMC8606189

[4] Nichterwitz S, Chen G, Aguila Benitez J. et al. Laser capture microscopy coupled with smart-seq2 for precise spatial transcriptomic profiling. *Nat Commun* 2016;7:12139. 10.1038/ncomms12139 27387371 PMC4941116

[5] Chen A, Liao S, Cheng M. et al. Spatiotemporal transcriptomic atlas of mouse organogenesis using DNA nanoball-patterned arrays. *Cell* 2022;185:1777–1792.e21. 10.1016/j.cell.2022.04.003 35512705

[6] Liu Y, Yang M, Deng Y. et al. High-spatial-resolution multi-omics sequencing via deterministic barcoding in tissue. *Cell* 2020;183:1665–1681.e18. 10.1016/j.cell.2020.10.026 33188776 PMC7736559

[7] Janesick A, Shelansky R, Gottscho AD. et al. High resolution mapping of the tumor microenvironment using integrated single-cell, spatial and in situ analysis. *Nat Commun* 2023;14:8353. 10.1038/s41467-023-43458-x 38114474 PMC10730913

[8] Ben-Chetrit N, Niu X, Swett AD. et al. Integration of whole transcriptome spatial profiling with protein markers. *Nat Biotechnol* 2023;41:788–93. 10.1038/s41587-022-01536-3 36593397 PMC10272089

[9] Liao S, Heng Y, Liu W. et al. Integrated spatial transcriptomic and proteomic analysis of fresh frozen tissue based on stereo-seq. *bioRxiv* 2023. 10.1101/2023.04.28.538364

[10] Zhang D, Deng Y, Kukanja P. et al. Spatial epigenome–transcriptome co-profiling of mammalian tissues. *Nature* 2023;616:113–22. 10.1038/s41586-023-05795-1 36922587 PMC10076218

[11] Liu Y, DiStasio M, Su G. et al. High-plex protein and whole transcriptome co-mapping at cellular resolution with spatial CITE-seq. *Nat Biotechnol* 2023;41:1405–9. 10.1038/s41587-023-01676-0 36823353 PMC10567548

[12] Jiang F, Zhou X, Qian Y. et al. Simultaneous profiling of spatial gene expression and chromatin accessibility during mouse brain development. *Nat Methods* 2023;20:1048–57. 10.1038/s41592-023-01884-1 37231265

[13] Vickovic S, Lötstedt B, Klughammer J. et al. SM-omics is an automated platform for high-throughput spatial multi-omics. *Nat Commun* 2022;13:795. 10.1038/s41467-022-28445-y 35145087 PMC8831571

[14] Wu X, Xu W, Deng L. et al. Spatial multi-omics at subcellular resolution via high-throughput in situ pairwise sequencing. *Nat Biomed Eng* 2024;8:872–89. 10.1038/s41551-024-01205-7 38745110

[15] Zhang D, Rubio Rodriguez-Kirby LA, Lin Y. et al. Spatial dynamics of mammalian brain development and neuroinflammation by multimodal tri-omics mapping. *bioRxiv* 2024. 10.1101/2024.07.28.605493

[16] Tian T, Zhang J, Lin X. et al. Dependency-aware deep generative models for multitasking analysis of spatial omics data. *Nat Methods* 2024;21:1501–13. 10.1038/s41592-024-02257-y PMC12410143 38783067

[17] Tang Z, Li Z, Hou T. et al. SiGra: single-cell spatial elucidation through an image-augmented graph transformer. *Nat Commun* 2023;14:5618. 10.1038/s41467-023-41437-w 37699885 PMC10497630

[18] Long Y, Ang KS, Li M. et al. Spatially informed clustering, integration, and deconvolution of spatial transcriptomics with GraphST. *Nat Commun* 2023;14:1155. 10.1038/s41467-023-36796-3 36859400 PMC9977836

[19] Hu J, Li X, Coleman K. et al. SpaGCN: integrating gene expression, spatial location and histology to identify spatial domains and spatially variable genes by graph convolutional network. *Nat Methods* 2021;18:1342–51. 10.1038/s41592-021-01255-8 34711970

[20] Zeng Y, Yin R, Luo M. et al. Identifying spatial domain by adapting transcriptomics with histology through contrastive learning. *Brief Bioinform* 2023;24:bbad048. 10.1093/bib/bbad048 36781228

[21] Liu T, Fang Z-Y, Li X. et al. Graph deep learning enabled spatial domains identification for spatial transcriptomics. *Brief Bioinform* 2023;24:bbad146. 10.1093/bib/bbad146 37080761

[22] Shi X, Zhu J, Long Y. et al. Identifying spatial domains of spatially resolved transcriptomics via multi-view graph convolutional networks. *Brief Bioinform* 2023;24:bbad278. 10.1093/bib/bbad278 37544658

[23] Si Z, Li H, Shang W. et al. SpaNCMG: improving spatial domains identification of spatial transcriptomics using neighborhood-complementary mixed-view graph convolutional network. *Brief Bioinform* 2024;25:bbae259. 10.1093/bib/bbae259 38811360 PMC11136618

[24] Wang T, Shu H, Hu J. et al. Accurately deciphering spatial domains for spatially resolved transcriptomics with stCluster. *Brief Bioinform* 2024;25:bbae329. 10.1093/bib/bbae329 38975895 PMC11771244

[25] Long Y, Ang KS, Sethi R. et al. Deciphering spatial domains from spatial multi-omics with SpatialGlue. *Nat Methods* 2024;21:1–10. 10.1038/s41592-024-02316-4 38907114 PMC11399094

[26] Benjamin K, Bhandari A, Kepple JD. et al. Multiscale topology classifies cells in subcellular spatial transcriptomics. *Nature* 2024;630:943–9. 10.1038/s41586-024-07563-1 38898271 PMC11208150

[27] Coleman K, Schroeder A, Li M. Unlocking the power of spatial omics with AI. *Nat Methods* 2024;21:1378–81. 10.1038/s41592-024-02363-x 39122938 PMC12023348

[28] Tang L . Spatially resolved multiomics. *Nat Methods* 2023;20:1871–1. 10.1038/s41586-023-06311-1 38057519

[29] Theodoris CV, Xiao L, Chopra A. et al. Transfer learning enables predictions in network biology. *Nature* 2023;618:616–24. 10.1038/s41586-023-06139-9 37258680 PMC10949956

[30] Yang F, Wang W, Wang F. et al. scBERT as a large-scale pretrained deep language model for cell type annotation of single-cell RNA-seq data. *Nat Mach Intell* 2022;4:852–66. 10.1038/s42256-022-00534-z

[31] Cui H, Wang C, Maan H. et al. scGPT: toward building a foundation model for single-cell multi-omics using generative AI. *Nat Methods* 2024;21:1470–80. 10.1038/s41592-024-02201-0 38409223

[32] Hao M, Gong J, Zeng X. et al. Large-scale foundation model on single-cell transcriptomics. *Nat Methods* 2024;21:1481–91. 10.1038/s41592-024-02305-7 38844628

[33] Wang L, Huang C, Wang M. et al. NeuroPred-PLM: an interpretable and robust model for neuropeptide prediction by protein language model. *Brief Bioinform* 2023;24:bbad077. 10.1093/bib/bbad077 36892166

[34] Cover T, Hart P. Nearest neighbor pattern classification. *IEEE Trans Inf Theory* 1967;13:21–7. 10.1109/TIT.1967.1053964

[35] Fabian P . Scikit-learn: machine learning in python. *J Mach Learn Res* 2011;12:2825. 10.5555/1953048.2078195

[36] Wang J, Ma A, Chang Y. et al. scGNN is a novel graph neural network framework for single-cell RNA-Seq analyses. *Nat Commun* 2021;12:1882. 10.1038/s41467-021-22197-x 33767197 PMC7994447

[37] Hao Y, Hao S, Andersen-Nissen E. et al. Integrated analysis of multimodal single-cell data. *Cell* 2021;184:3573–3587.e29. 10.1016/j.cell.2021.04.048 34062119 PMC8238499

[38] Gayoso A, Steier Z, Lopez R. et al. Joint probabilistic modeling of single-cell multi-omic data with totalVI. *Nat Methods* 2021;18:272–82. 10.1038/s41592-020-01050-x 33589839 PMC7954949

[39] Ashuach T, Gabitto MI, Koodli RV. et al. MultiVI: deep generative model for the integration of multimodal data. *Nat Methods* 2023;20:1222–31. 10.1038/s41592-023-01909-9 37386189 PMC10406609

[40] Argelaguet R, Arnol D, Bredikhin D. et al. MOFA+: a statistical framework for comprehensive integration of multi-modal single-cell data. *Genome Biol* 2020;21:1–17. 10.1186/s13059-020-02015-1 PMC7212577 32393329

[41] Minoura K, Abe K, Nam H. et al. A mixture-of-experts deep generative model for integrated analysis of single-cell multiomics data. *Cell Rep Methods* 2021;1:100071. 10.1016/j.crmeth.2021.100071 35474667 PMC9017195

[42] Ghazanfar S, Guibentif C, Marioni JC. Stabilized mosaic single-cell data integration using unshared features. *Nat Biotechnol* 2024;42:284–92. 10.1038/s41587-023-01766-z 37231260 PMC10869270

