# Supplementary material for: spaLLM: enhancing spatial domain analysis in multi-omics data through large language model integration
Source: Brief Bioinform. 2025 Jul 3;26(4):bbaf304. doi: 10.1093/bib/bbaf304 (PMC12224616; doi:10.1093/bib/bbaf304)
Supplement: Supplementary_materials_bbaf304 [file supplementary_materials_bbaf304.docx]

**Supplementary Figures**

**
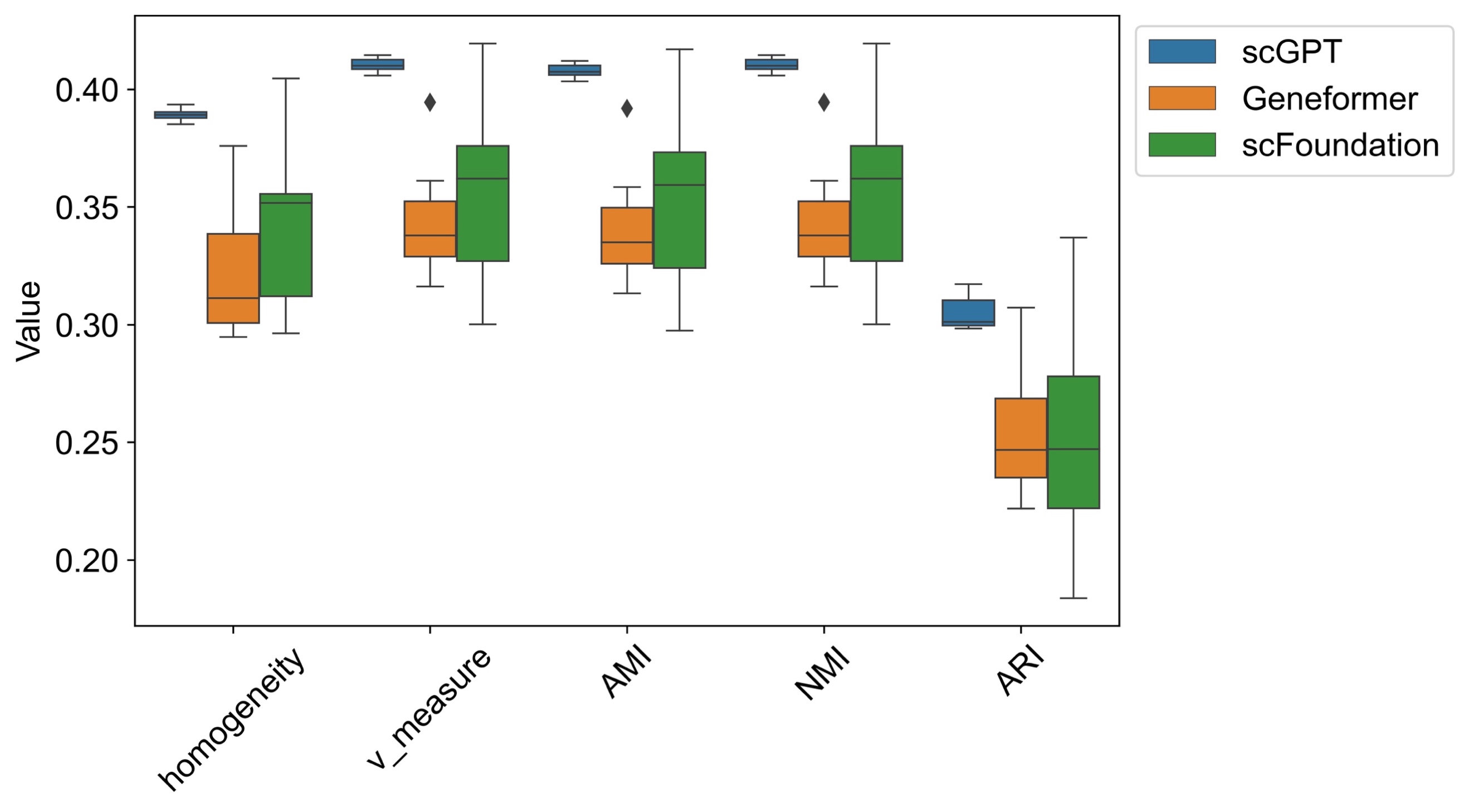
**

**Figure S1.** A comparison of spaLLM using embeddings from different large language models (GeneFormer and scFoundation). Box plots depict the distributions of five evaluation metrics—homogeneity, v_measure, AMI, NMI, and ARI—across seven independent training runs conducted with different iteration counts, illustrating the overall superior and more stable performance of scGPT relative to the other models. In each plot, the central line denotes the median, the box represents the interquartile range, and the whiskers extend to 1.5 times the interquartile range.

**Figure S2.** Evaluating the Robustness of the Attention Calculation under Different Initialisation Parameters. Specifically, we compared three different initialisation methods: Xavier Uniform (used in spaLLM), Xavier Normal, and Orthogonal initialisations. (A) The violin plot of the attention weights between the LLM embedding encoded with spatial information and the omic embedding using Xavier Uniform. (B) The violin plot of the attention weights between the LLM embedding encoded with feature information and the omic embedding using Xavier Uniform. (C) The violin plot of the attention weights between the LLM embedding encoded with spatial information and the omic embedding using Xavier Normal. (D) The violin plot of the attention weights between the LLM embedding encoded with feature information and the omic embedding using Xavier Normal. (E) The violin plot of the attention weights between the LLM embedding encoded with spatial information and the omic embedding using Orthogonal. (F) The violin plot of the attention weights between the LLM embedding encoded with feature information and the omic embedding using Orthogonal.


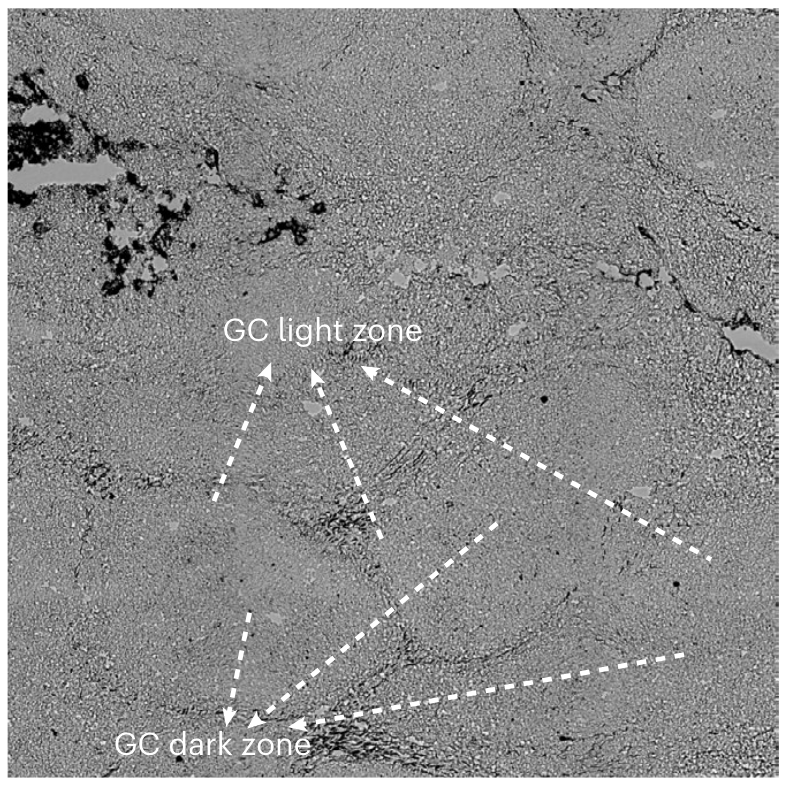


**Figure S3.** The histological annotation of the Spatial-CITE-seq human tonsil dataset from Tian et al [1]. The GC light and dark zones are marked.

**Figure S4.** Spatial domains detected by spaLLM in clusters 6 (A) and 7 (B).

**Figure S5.** (A) The violin plot of the attention weights between the LLM embedding encoded with spatial information and the omic embedding. (B) The violin plot of the attention weights between the LLM embedding encoded with feature information and the omic embedding

**References**

1. Tian T, Zhang J, Lin X et al. Dependency-aware deep generative models for multitasking analysis of spatial omics data. Nature Methods 2024:1-13.
